# Ravulizumab in neuromyelitis optica spectrum disorder: first clinical experience in Chile

**DOI:** 10.3389/fimmu.2026.1932448

**Published:** 2026-10-06

**Authors:** Tamara Santibáñez, José Miguel Valdés, César Romero, Manuel Fruns, Lorna Galleguillos

**Affiliations:** 1 Neuroimmunology Unit, Department of Neurology, Clínica Alemana de Santiago, Santiago, Chile; 2 Hospital Dra. Eloísa Díaz Inzunza, La Florida, Santiago, Chile; 3 Clínica RedSalud, Santiago, Chile; 4 Hospital Clínico Universidad de Chile, Santiago, Chile; 5 Clínica Universidad de los Andes, Santiago, Chile; 6 Clínica Las Condes, Santiago, Chile; 7 Clínica Dávila, Santiago, Chile

**Keywords:** AQP4-IgG, Chile, complement inhibitor, neuromyelitis optica spectrum disorder, ravulizumab, real-world evidence

## Abstract

**Background:**

Neuromyelitis optica spectrum disorder (NMOSD) is a neuroimmunological astrocytopathy mediated by anti-aquaporin-4 (AQP4-IgG) antibodies that trigger complement-dependent cytotoxicity and activation of the terminal complement cascade in the central nervous system. Ravulizumab, a long-acting terminal complement C5 inhibitor, received regulatory approval in Chile in January 2025; the seven patients reported here represent the entire nationally treated cohort to date.

**Objectives:**

To describe the first clinical experience with ravulizumab in NMOSD at three Chilean centers (Clínica Alemana de Santiago, a private center, Clínica Dávila, a private center, and Hospital Clínico Universidad de Chile, a public university hospital).

**Methods:**

Retrospective, multicenter cohort study of patients with NMOSD fulfilling the 2015 International Panel for NMO Diagnosis (IPND) consensus criteria who were receiving ravulizumab. Demographic and clinical characteristics, AQP4-IgG status, baseline and follow-up Expanded Disability Status Scale (EDSS) scores, treatment indication, relapses and adverse events were analyzed through medical chart review.

**Results:**

Seven patients (100% female) were included, with a mean age at disease onset of 51.0 years (range 38–69). AQP4-IgG was positive in all patients (100%), confirmed by cell-based assay. The most common presentation over the disease course was longitudinally extensive transverse myelitis (LETM; 7/7); four patients (57.1%) also developed optic neuritis. Median baseline EDSS was 6.0. Five patients had previously failed rituximab, one discontinued rituximab because of active cancer, and one was treatment naïve. Over a median follow-up of 15 months (range 4–24) on ravulizumab, no clinical relapses were observed (0/7 patients), compared with approximately 4.5 relapses expected across the cohort’s person-time had the pre-treatment relapse rate continued unchanged (observed-vs-expected analysis; see Results). EDSS declined from a baseline median of 6.0 to 2.5 (median within-patient change 1.5 points, IQR 1.0–4.0); because baseline EDSS in several patients was recorded during or shortly after an acute attack, this decrease likely reflects post-attack recovery in addition to any disability-modifying effect, and three patients remained at EDSS ≥5. Headache was the only adverse event (7/7), mild to moderate and self-limited.

**Conclusions:**

In this real-world Chilean cohort, ravulizumab was associated with complete relapse suppression over a median follow-up of 15 months and was well tolerated. These preliminary, uncontrolled observations are consistent with the relapse-prevention efficacy reported in pivotal trials and with regional consensus guidance on its use after rituximab failure, and they underscore the need to expand equitable access to complement-targeted therapies within the Chilean healthcare system.

## Introduction

Neuromyelitis optica spectrum disorder (NMOSD) was first described by Eugène Devic in 1894 as the association of bilateral optic neuritis and acute myelitis (1). The discovery of the NMO-IgG autoantibody by Lennon et al. in 2004 (2), and its identification as an anti-aquaporin-4 (AQP4) antibody, established NMOSD as a distinct autoimmune astrocytopathy (3, 4).

AQP4 is the most abundant water channel in the central nervous system (CNS), expressed predominantly in astrocytic end-feet surrounding blood vessels and at the ependymal surface. The anatomical regions with the highest AQP4 expression—optic nerve, central spinal cord, area postrema, periependymal regions of the third and fourth ventricles, and diencephalon—correspond to the classic topographic sites of the disease.

Binding of AQP4-IgG to astrocytic channels triggers three principal effector mechanisms (3, 5). First, complement-dependent cytotoxicity (CDC) involves activation of the classical complement pathway (C1q → C3 → C5) and formation of the membrane attack complex (MAC, C5b-9); this mechanism is the direct target of C5 inhibitors (eculizumab and ravulizumab). Second, antibody-dependent cellular cytotoxicity (ADCC) is mediated by natural killer cells and granulocytes. Third, the release of pro-inflammatory cytokines—particularly interleukin-6 (IL-6)—perpetuates the differentiation of AQP4-IgG-producing plasmablasts and disrupts the blood–brain barrier; this pathway is the target of satralizumab.

NMOSD is a rare disease, with an estimated global prevalence of 0.5–4 cases per 100,000 inhabitants and higher rates in populations of African and East Asian descent. It predominantly affects women (female-to-male ratio approximately 9:1) and typically presents between the fourth and fifth decades of life, although pediatric and late-onset cases occur (6).

Clinically, NMOSD is characterized by three core syndromes: (1) optic neuritis, which may be bilateral or sequential and typically involves more than 50% of the optic nerve length, with posterior and chiasmatic predominance; (2) longitudinally extensive transverse myelitis (LETM), spanning more than three vertebral segments, with the classic ‘H-sign’ on MRI; and (3) the area postrema syndrome, characterized by intractable hiccups and/or nausea and vomiting lasting more than 48 hours without a gastrointestinal cause (7, 8).

The principal MRI findings include dorsal brainstem lesions adjacent to the fourth ventricle involving the area postrema, contiguous central-cord lesions, and large confluent or tumefactive cerebral white-matter lesions that may follow white-matter tracts (7, 8).

The discovery of AQP4-IgG substantially transformed both diagnosis and the development of targeted therapies. Among detection methods, cell-based assays (CBA) offer the best sensitivity and specificity and should use fixed or live cells expressing full-length, conformationally intact human AQP4, with sham-transfected or non-transfected cells as negative substrates (9, 10). Fixed-cell CBA kits are more widely available and easier to process for large volumes but are less sensitive; live-cell CBAs are more sensitive and are considered the gold standard, yet are available at only a small number of centers worldwide (9, 10).

The first diagnostic criteria were proposed by Wingerchuk et al. in 1999 (11). In 2015, the International Panel for NMO Diagnosis (IPND) established the current international consensus diagnostic criteria, which stratify diagnosis by AQP4-IgG serostatus and permit a diagnosis after a single core clinical syndrome in seropositive patients (12). Subsequent reviews have reaffirmed these criteria and summarized the expanding therapeutic landscape (13, 14).

Acute treatment is guided by clinical severity and comprises corticosteroids, immunoglobulin and/or plasma exchange; earlier initiation improves short- and long-term functional outcomes (15). For maintenance, rituximab (anti-CD20) is widely used at doses of 500–1000 mg every six months (16). Since 2019, four approved monoclonal antibodies—eculizumab, satralizumab, inebilizumab and ravulizumab—have demonstrated marked reductions in the annualized relapse rate (ARR) in AQP4-IgG-positive patients in pivotal phase III trials, with a recent systematic review and meta-analysis confirming reduced relapse risk and disability progression across this drug class (17–21). In Chile, only two of these therapies have been approved by the Instituto de Salud Pública (ISP): satralizumab (2020) and ravulizumab (2025). Owing to limitations in healthcare coverage, very few patients have accessed them to date. Here we report the first seven cases treated with ravulizumab for NMOSD in Chile.

## Materials and methods

### Study design and population

This was a retrospective, multicenter cohort study conducted at three Chilean neurological centers: Clínica Alemana de Santiago (private), Clínica Dávila (private), and Hospital Clínico Universidad de Chile (public university hospital). All patients who fulfilled the 2015 IPND consensus criteria for NMOSD (12) and who were receiving ravulizumab at the time of data collection were included. These seven patients represent the entire cohort treated with ravulizumab nationwide to date. Two patients initiated ravulizumab before formal ISP approval, through named-patient/early-access (compassionate-use) arrangements; the remainder started after approval. One patient was referred from Clínica Las Condes to Clínica Dávila for ravulizumab infusion due to insurance/coverage reasons.

### Data collection

Demographic data, comorbidities, clinical phenotype, AQP4-IgG status and titer, prior therapies, treatment indication, Expanded Disability Status Scale (EDSS) scores (22) at baseline and at the most recent follow-up visit, relapses during ravulizumab treatment, and adverse events were collected through medical chart review.

### Definition of relapse

A relapse was defined as a new or worsening neurological symptom attributable to NMOSD, lasting more than 24 hours, with an objective clinical and/or radiological correlate, and separated from a preceding event by at least 30 days. The acute attack prompting initiation of ravulizumab in the treatment-naïve patient was counted as a relapse.

### Antibody testing

AQP4-IgG positivity was confirmed by CBA in all patients: fixed-cell CBA (n = 4) or live-cell CBA performed at Mayo Clinic (n = 3). Seronegative cases and patients positive only for MOG-IgG were excluded.

### Treatment protocol

Ravulizumab was administered intravenously according to the standard weight-based dosing protocol established in the CHAMPION-NMOSD trial (20): a loading dose on day 1, followed by a first maintenance dose on day 15 and maintenance doses every eight weeks thereafter. All patients received meningococcal vaccination (serogroups A, C, W, Y and B) before or at treatment initiation, per current guidance for patients receiving terminal complement inhibitors (23).

### Outcomes

The primary outcome was the occurrence of clinical relapses during ravulizumab treatment. Secondary outcomes were the change in EDSS from baseline to last follow-up and the safety profile.

### Statistical analysis

Continuous variables are summarized as mean ± standard deviation or as median (interquartile range, IQR, defined as the 25th–75th percentile), according to distribution; categorical variables are summarized as counts and percentages. Disease duration before ravulizumab initiation was calculated as the interval between the first documented NMOSD event and the date of ravulizumab initiation. For each patient, the pre-treatment annualized relapse rate was calculated as the number of relapses documented in the approximately 24 months preceding ravulizumab initiation, divided by that observation period (**Table 1**). The number of relapses expected during ravulizumab treatment, had the pre-treatment rate continued unchanged, was calculated for each patient as the product of their individual pre-treatment annualized relapse rate and their individual observed person-time on ravulizumab, summed across the cohort (observed-versus-expected analysis). A pre-specified sensitivity analysis was performed excluding Patient 2, whose only pre-treatment event was the index attack that prompted first-line ravulizumab initiation as a treatment-naïve patient and is therefore not methodologically equivalent to a true pre-treatment relapse rate in the other six, previously treated patients. All analysis were descriptive and exploratory; no formal hypothesis testing was performed given the sample size. Statistical analysis were performed using [software and version to be confirmed by the corresponding author]. The data cutoff for this analysis was [date to be confirmed by the corresponding author].

**Table 1:** Prior therapy, relapses, and safety outcomes.

Variable	Value
Rituximab with therapeutic failure, n (%)	5/7 (71.4%)
Rituximab discontinued due to active cancer, n (%)	1/7 (14.3%)
Treatment-naïve (ravulizumab first-line), n (%)	1/7 (14.3%)
Prior satralizumab, n (%)	1/7 (14.3%)
Total documented relapses, median (range)	3 (1–8)
Relapses in the ~24 months before ravulizumab, median (range)	1 (1–2)
Relapses during ravulizumab, n (%)	0/7 (0.0%)
Median follow-up (months)	15 [range 4–24]
Headache (mild–moderate, self-limited), n (%)	7/7 (100.0%)
Meningococcal infection, n (%)	0/7 (0.0%)
Serious adverse events, n (%)	0/7 (0.0%)

All patients received meningococcal vaccination before ravulizumab initiation. Headache was mild to moderate and self-limited in all cases, not requiring drug discontinuation.

### Ethical considerations

The study was conducted in accordance with the principles of the Declaration of Helsinki, and all patients provided written informed consent for the publication of their clinical data. The study was reviewed and approved by the institutional research ethics committee of the coordinating center (Comité de Ética e Investigación, Universidad del Desarrollo; protocol version 1.0, dated 10 November 2024; approval granted 12 December 2024).

## Results

### Baseline characteristics

Seven patients were included, all female (100%), with a mean current age of 60.1 ± 12.3 years (range 43–75) and a mean age at disease onset of 51.0 ± 11.6 years (range 38–69). Median disease duration before ravulizumab initiation was 7.0 years (IQR 3.0–14.0; range <1–16); the treatment-naïve patient (P2) had a disease duration of less than one year. AQP4-IgG was positive in all patients; three (42.9%) had quantitative titers ranging from 1:1,000 to 1:100,000 by live-cell CBA at Mayo Clinic. Over the disease course, clinical presentations included LETM in all seven patients (100%) and optic neuritis in four (57.1%); brain MRI lesions were present in one patient (14.3%). A high prevalence of associated autoimmune comorbidities was observed (**Table 2**). Individual patient data are presented in **Table 3**.

**Table 2:** Baseline demographic and clinical characteristics of the cohort (n = 7).

Variable	Value
Current age (years), mean ± SD	60.1 ± 12.3 [range 43–75]
Female sex, n (%)	7/7 (100.0%)
African or Asian descent, n	0/7
Age at onset (years), mean ± SD	51.0 ± 11.6 [range 38–69]
Disease duration (years), median (IQR)	7.0 (3.0–14.0) [range <1–16]
AQP4-IgG positive, n (%)	7/7 (100.0%)
— Fixed-cell assay (CBA), n (%)	4/7 (57.1%)
— Live-cell assay (CBA), n (%)	3/7 (42.9%) — titres 1:1,000 to 1:100,000
LETM, n (%)	7/7 (100.0%)
Optic neuritis, n (%)	4/7 (57.1%)
Brain lesions, n (%)	1/7 (14.3%)
Comorbidities	Hashimoto/hypothyroidism (2), Sjögren (1), rheumatoid arthritis (1), type 1 diabetes (1), SLE (1), fibromyalgia (1), mood disorder (1), hypertension (1), active cancer (1)

CBA, cell-based assay; LETM, longitudinally extensive transverse myelitis; SLE, systemic lupus erythematosus. Comorbidities are not mutually exclusive.

**Table 3:** Individual patient characteristics, prior therapies, and outcomes.

Pt	Sex/age	Clinical phenotype (year)	EDSS base	AQP4-IgG (method, titre)	Prior therapy/reason	Relapses total (n)	Relapses prior ~24 mo	Ravu start	F-up (mo)	EDSS f-up
P1	F/49	ON (2015) LETM (2017, 2023)	3.0	Positive (qual.), fixed CBA	Rituximab/failure	3	1	Mar 2025	~15	2.5
P2	F/43	LETM (2024, index attack)	6.0	Positive (qual.), fixed CBA	Naïve/first-line	1	1	Dec 2024	~18	2.5
P3	F/60	LETM ×4 (2009–2013) ON ×3 (2018–2022) LETM (2024)	6.5	Live CBA 1:1,000 (Mayo)	Rituximab/failure	8	1	Feb 2025	~16	2.5
P4	F/53	LETM (2021–2024)	6.0	Live CBA 1:1,000 (Mayo)	Rituximab/failure	1	1	Jun 2024	~24	2.0
P5	F/75	LETM (2020, 2022) ON (2025)	6.0	Live CBA 1:100,000 (Mayo)	Rituximab/failure	3	1	Jan 2026	~4	5.0
P6	F/72	LETM (2011, 2012, 2018, 2023) ON (2024)	7.0	Positive (qual.), fixed CBA	Rituximab → satralizumab/failure	5	2	Sep 2025	~9	6.0
P7	F/69	LETM (2018, 2021, 2025)	7.0	Positive (qual.), fixed CBA	Rituximab/discontinued (active cancer)	3	1	Apr 2025	~14	5.5

CBA, cell-based assay; EDSS, Expanded Disability Status Scale; F-up, follow-up; LETM, longitudinally extensive transverse myelitis; ON, optic neuritis; qual., qualitative; Ravu, ravulizumab. Total relapses counted per the relapse definition in Methods; relapses in the ~24 months before ravulizumab are approximate, derived from documented event years. Baseline EDSS was recorded at the visit closest to ravulizumab initiation and may reflect an acute attack in some patients.

### Prior therapy and indication for ravulizumab

Five patients (71.4%) had documented therapeutic failure to rituximab 1 g every 6 months before initiating ravulizumab (**Table 1**). One patient discontinued rituximab because of active cancer, and one was treatment-naïve and started ravulizumab as first-line therapy during an acute LETM attack. One patient had also received satralizumab for four years sequentially after rituximab failure before switching to ravulizumab because of treatment failure.

### Individual patient profiles

Detailed individual data are presented in **Table 3**. The most common prior therapy was rituximab (6/7 patients). Applying the relapse definition above, the total number of documented relapses ranged from 1 to 8 per patient (median 3), and most patients had experienced one relapse in the approximately 24 months preceding ravulizumab (P6 had two). Patient 3 had the highest disease burden, with eight documented relapses over 16 years. Patient 2, the only treatment-naïve individual, began ravulizumab during an acute LETM attack, which was counted as a relapse.

### Efficacy outcomes

During a median follow-up of 15 months (range 4–24) on ravulizumab, no clinical relapses were recorded (0/7 patients). Total exposure on ravulizumab was 100 patient-months (8.3 person-years). Using each patient’s individual pre-ravulizumab relapse rate (relapses in the ~24 months before initiation; **Figure 1**) projected over their own observed person-time on ravulizumab, approximately 4.5 relapses would have been expected had the pre-treatment rate continued unchanged; zero were observed. A pre-specified sensitivity analysis excluding Patient 2 (whose only pre-treatment event was the index attack that prompted first-line ravulizumab initiation as a treatment-naïve patient) yielded an expected 3.8 relapses among the remaining six previously treated patients, consistent with the primary estimate. Given the small sample, short and heterogeneous follow-up, and the approximate nature of the pre-treatment relapse-rate estimate, this observed-versus-expected comparison is exploratory and hypothesis-generating rather than a formal test of efficacy; relapse prevention, rather than a specific magnitude of annualized-relapse-rate reduction, is the outcome these data support. EDSS decreased from a baseline median of 6.0 (range 3.0–7.0) to 2.5 (range 2.0–6.0) at last follow-up. The median within-patient EDSS change was 1.5 points (IQR 1.0–4.0), with individual reductions of 0.5, 3.5, 4.0, 4.0, 1.0, 1.0 and 1.5 points. Three patients (P5, P6 and P7) remained at EDSS ≥5 at follow-up. Baseline EDSS was recorded at the visit closest to ravulizumab initiation, which in several cases coincided with or shortly followed an acute attack; accordingly, part of the observed EDSS decrease likely reflects post-attack recovery rather than a direct effect of ravulizumab on fixed disability, and these changes should be interpreted with caution. Outcomes are summarized in **Table 3**.

**Figure 1:**
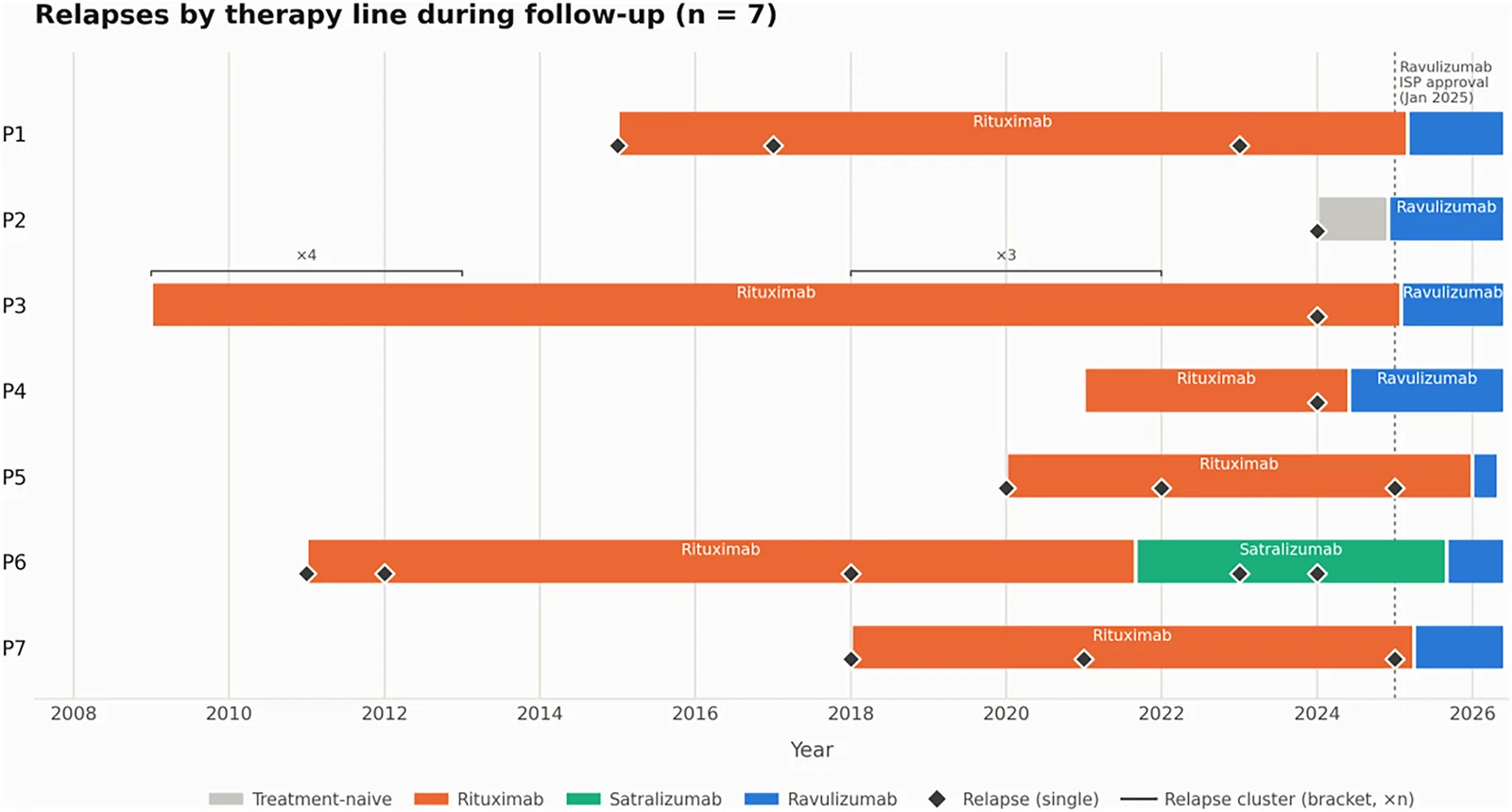
Relapse timing relative to line of therapy during follow-up (n = 7). Horizontal bars show the therapy category in use for each patient (colored by drug) leading up to, and including, ravulizumab treatment; diamonds mark individual documented relapses and bracketed spans mark clustered relapses reported only as an event count within a date range (Patient 3). No relapses were recorded during ravulizumab treatment in any patient (0/7). Exact start dates of prior therapies (other than ravulizumab) were not systematically available in the medical record; bars are anchored to the first documented disease event and should be read as the therapy line in use, not as continuous confirmed dosing throughout. For Patient 6, the rituximab-to-satralizumab segment boundary is back-calculated from the ~4-year satralizumab exposure reported in Results, ending at the September 2025 switch to ravulizumab; the exact calendar start date of satralizumab was not otherwise specified in the medical record.

### Safety

Headache was the only adverse event, occurring in all seven patients (100%); it was consistently mild to moderate, self-limited, and did not require treatment discontinuation. No meningococcal infections or serious adverse events were documented during follow-up (**Table 1**).

## Discussion

In this real-world Chilean cohort of seven AQP4-IgG-positive NMOSD patients, ravulizumab was associated with complete relapse suppression during a median follow-up of 15 months (range 4–24). Using the cohort’s own pre-treatment relapse rate, the observed-versus-expected analysis (Results) suggested that approximately 4.5 relapses would otherwise have occurred over the same person-time; the small sample size, low absolute event rate, and short follow-up limit the precision of this estimate, and it should be read as exploratory rather than a formal test of efficacy. The pivotal CHAMPION-NMOSD trial reported a 98.6% reduction in relapse risk (time to first adjudicated relapse) relative to an external placebo comparator drawn from the earlier PREVENT-NMOSD trial (CHAMPION-NMOSD itself had no internal placebo arm), in a population with a substantially higher baseline ARR than the present cohort; a direct comparison of effect sizes between the two studies is therefore not appropriate, and our findings are best regarded as qualitatively consistent with, rather than confirmatory of, the relapse-prevention signal reported in CHAMPION-NMOSD (20). Individual real-world case reports of ravulizumab in AQP4-IgG-positive NMOSD have similarly described complete relapse suppression sustained over multi-year follow-up, corroborating this relapse-prevention signal outside the trial setting (24).

Median EDSS improved from 6.0 to 2.5; however, the median within-patient improvement was only 1.5 points (IQR 1.0–4.0), and three patients (P5, P6 and P7) remained at EDSS ≥5. Because a terminal C5 inhibitor prevents relapses but does not reverse established disability, and because baseline EDSS may have been recorded during or shortly after an acute attack, part of this decrease most likely reflects post-attack recovery rather than a direct disability-modifying effect. We therefore frame the primary benefit of ravulizumab as relapse prevention rather than improvement of fixed disability. This is particularly relevant given that 71.4% of patients had previously failed rituximab—widely used first-line in Latin America owing to its availability and lower cost. Whether each of these relapses occurred despite adequately dosed rituximab with confirmed B-cell depletion, or during under-dosing or treatment gaps, could not be systematically reconstructed for all patients and is a limitation of this series (see Limitations); with that caveat, these real-world observations are consistent with the value of ravulizumab as an option after rituximab failure, in line with regional consensus recommendations (25).

The safety profile was consistent with CHAMPION-NMOSD. Headache occurred in all patients but was mild to moderate and self-limited. No serious adverse events—including meningococcal infection, the most critical safety concern with complement inhibition—were recorded; systematic pre-treatment meningococcal vaccination was implemented per protocol, though no causal attribution can be drawn from this observation given the small sample size (20). These observations underscore the importance of standardized meningococcal vaccination as part of the management protocol.

Mechanistically, ravulizumab offers advantages over eculizumab. As a recycled antibody with a modified neonatal Fc receptor (FcRn) binding domain, it has a substantially prolonged half-life (~50 days versus ~11 days for eculizumab), enabling an every-eight-week maintenance schedule (14). This may improve adherence and quality of life compared with biweekly infusions, which is particularly relevant in a country with limited infusion infrastructure such as Chile.

This report also reflects a broader regional challenge: access to evidence-based NMOSD therapies remains markedly inequitable. In Chile, only two of the four approved monoclonal antibodies (satralizumab and ravulizumab) have ISP registration, and both are accessible almost exclusively through private insurance, named-patient/compassionate-access programs, or the military healthcare system. This creates a treatment gap for patients dependent on the public health system (FONASA – national health system), who constitute most of the population. Expanding coverage and developing health-technology-assessment pathways for these therapies should be a priority for policymakers.

## Limitations

- Small sample size (n = 7), which limits statistical power and generalizability.
- Retrospective, non-controlled design without a comparator group.
- The comparison with pivotal-trial effect sizes (e.g., CHAMPION-NMOSD) is qualitative only: the trial population had a substantially higher baseline annualized relapse rate, and its comparator was an external, historical placebo group (from PREVENT-NMOSD) rather than a concurrent internal placebo arm; the low pre-treatment event rate in this cohort further limits the power of the within-cohort observed-versus-expected analysis to demonstrate efficacy.
- Heterogeneous follow-up duration (range 4–24 months), reflecting the recent availability of ravulizumab in Chile.
- Heterogeneous AQP4-IgG methodology (qualitative fixed-cell CBA vs quantitative live-cell CBA), precluding correlation between antibody titer and clinical response.
- Baseline EDSS timing relative to the last attack was not uniformly available, limiting interpretation of the observed EDSS changes.
- Concurrent therapy, dose/interval, and (for rituximab) B-cell depletion status at the time of each pre-ravulizumab relapse could not be systematically reconstructed for all patients, limiting the ability to distinguish relapse on adequately dosed therapy from relapse during under-treatment or a treatment gap.

Notwithstanding these limitations, the results are consistent with international evidence and provide clinically relevant data from a Latin American population that has been under-represented in pivotal trials.

## Conclusions

NMOSD has undergone a diagnostic and therapeutic transformation over the past two decades, and targeted therapies have radically improved the prognosis of AQP4-IgG-positive disease. This report describes the first real-world experience with ravulizumab in Chile, demonstrating complete relapse suppression and a favorable safety profile during a median follow-up of 15 months. Observed EDSS changes should be interpreted cautiously and are most consistent with post-attack recovery combined with relapse prevention rather than reversal of fixed disability. Current challenges include achieving gold-standard antibody detection (live-cell CBA) and ensuring equitable access to approved therapies across the public and private healthcare sectors.

## Data Availability

The raw data supporting the conclusions of this article will be made available by the authors, without undue reservation.

## References

[1] Devic E . Myélite subaiguë compliquée de névrite optique. Bull Med (Paris). (1894) 8:1033–4.

[2] Lennon VA Wingerchuk DM Kryzer TJ Pittock SJ Lucchinetti CF Fujihara K . A serum autoantibody marker of neuromyelitis optica: distinction from multiple sclerosis. Lancet. (2004) 364:2106–12. doi: 10.1016/s0140-6736(04)17551-x 15589308

[3] Jarius S Paul F Weinshenker BG Levy M Kim HJ Wildemann B . Neuromyelitis optica. Nat Rev Dis Primers. (2020) 6:85. doi: 10.1038/s41572-020-0214-9 33093467

[4] Lucchinetti CF Guo Y Popescu BF Fujihara K Itoyama Y Misu T . The pathology of an autoimmune astrocytopathy: lessons learned from neuromyelitis optica. Brain Pathol. (2014) 24:83–97. doi: 10.1111/bpa.12099 24345222 PMC3905574

[5] Hinson SR Lennon VA Pittock SJ . Autoimmune AQP4 channelopathies and neuromyelitis optica spectrum disorders. Handb Clin Neurol. (2016) 133:377–403. doi: 10.1016/B978-0-444-63432-0.00021-9 27112688

[6] Hor JY Asgari N Nakashima I Broadley SA Leite MI Kissani N . Epidemiology of neuromyelitis optica spectrum disorder and its prevalence and incidence worldwide. Front Neurol. (2020) 11:501. doi: 10.3389/fneur.2020.00501 32670177 PMC7332882

[7] Kim HJ Paul F Lana-Peixoto MA Tenembaum S Asgari N Palace J . MRI characteristics of neuromyelitis optica spectrum disorder: an international update. Neurology. (2015) 84:1165–73. doi: 10.1212/wnl.0000000000001367 25695963 PMC4371410

[8] Shosha E Dubey D Palace J Nakashima I Jacob A Fujihara K . Area postrema syndrome: frequency, criteria, and severity in AQP4-IgG-positive NMOSD. Neurology. (2018) 91:e1642–51. doi: 10.1212/WNL.0000000000006392 30258024 PMC6205685

[9] Ye XF Huang ZP Li MM Liu SF Huang WL Hamud AMS . Update on aquaporin-4 antibody detection: the early diagnosis of neuromyelitis optica spectrum disorders. Mult Scler Relat Disord. (2024) 90:105175. doi: 10.1016/j.msard.2024.105803 39128164

[10] Waters P Reindl M Saiz A Schanda K Tuller F Kral V . Multicentre comparison of a diagnostic assay: aquaporin-4 antibodies in neuromyelitis optica. J Neurol Neurosurg Psychiatry. (2016) 87:1005–15. doi: 10.1136/jnnp-2015-312601 27113605 PMC5013123

[11] Wingerchuk DM Hogancamp WF O’Brien PC Weinshenker BG . The clinical course of neuromyelitis optica (Devic’s syndrome). Neurology. (1999) 53:1107–14. doi: 10.1212/wnl.53.5.1107 10496275

[12] Wingerchuk DM Banwell B Bennett JL Cabre P Carroll W Chitnis T . International consensus diagnostic criteria for neuromyelitis optica spectrum disorders. Neurology. (2015) 85:177–89. doi: 10.1212/wnl.0000000000001729 26092914 PMC4515040

[13] Cacciaguerra L Flanagan EP . Updates in NMOSD and MOGAD diagnosis and treatment. Neurol Clin. (2024) 42:77–114. doi: 10.1016/j.ncl.2023.06.009 37980124 PMC10658081

[14] Holian AH Weinshenker BG . Emerging role of targeted monoclonal antibodies in neuromyelitis optica spectrum disorders. BioDrugs. (2025) 39:573–89. doi: 10.1007/s40259-025-00729-x 40506618 PMC12185636

[15] Bonnan M Valentino R Debeugny S Merle H Fergé JL Mehdaoui H . Short delay to initiate plasma exchange is the strongest predictor of outcome in severe attacks of NMO. J Neurol Neurosurg Psychiatry. (2018) 89:346–51. doi: 10.1136/jnnp-2017-316286 29030418

[16] Tahara M Oeda T Okada K Kiriyama T Ochi K Maruyama H . Safety and efficacy of rituximab in neuromyelitis optica spectrum disorders (RIN-1 study): a multicentre, randomised, double-blind, placebo-controlled trial. Lancet Neurol. (2020) 19:298–306. doi: 10.1016/s1474-4422(20)30066-1 32199095

[17] Pittock SJ Berthele A Fujihara K Kim HJ Levy M Palace J . Eculizumab in aquaporin-4-positive neuromyelitis optica spectrum disorder (PREVENT). N Engl J Med. (2019) 381:614–25. doi: 10.1056/nejmoa1900866 31050279

[18] Yamamura T Kleiter I Fujihara K Palace J Greenberg B Zakrzewska-Pniewska B . Trial of satralizumab in neuromyelitis optica spectrum disorder. N Engl J Med. (2019) 381:2114–24. doi: 10.1056/nejmoa1901747 31774956

[19] Cree BAC Bennett JL Kim HJ Weinshenker BG Pittock SJ Wingerchuk DM . Inebilizumab for the treatment of neuromyelitis optica spectrum disorder (N-MOmentum): a double-blind, randomised placebo-controlled phase 2/3 trial. Lancet. (2019) 394:1352–63. doi: 10.1016/s0140-6736(19)31817-3 31495497

[20] Pittock SJ Barnett M Bennett JL Berthele A de Sèze J Levy M . Ravulizumab in aquaporin-4-positive neuromyelitis optica spectrum disorder (CHAMPION-NMOSD). Ann Neurol. (2023) 93:1053–68. doi: 10.1002/ana.26626 36866852

[21] de Melo PS de Oliveira Costa E Barreto Ribeiro CM Pereira de Jesus PA Lordelo San Martin D . Monoclonal antibodies in neuromyelitis optica spectrum disorder: a systematic review and meta-analysis. BMC Neurol. (2026) 26:185. doi: 10.1186/s12883-026-04736-5 41703474 PMC13015146

[22] Kurtzke JF . Rating neurologic impairment in multiple sclerosis: an expanded disability status scale (EDSS). Neurology. (1983) 33:1444–52. doi: 10.1212/WNL.33.11.1444 6685237

[23] Mbaeyi SA Bozio CH Duffy J Rubin LG Hariri S Stephens DS . Meningococcal vaccination: recommendations of the Advisory Committee on Immunization Practices, United States, 2020. MMWR Recomm Rep. (2020) 69:1–41. doi: 10.15585/mmwr.rr6909a1 33417592 PMC7527029

[24] Akkawi N Hassabo Mohamed E Shatila A . Ravulizumab for relapse prevention in AQP4-IgG-positive neuromyelitis optica spectrum disorder: a 2-year follow-up case report. Front Med (Lausanne). (2026) 13:1827687. doi: 10.3389/fmed.2026.1827687 42369157 PMC13303217

[25] Carnero Contentti E Rojas JI Cristiano E Marques VD Flores-Rivera J Lana-Peixoto M . Latin American consensus recommendations for management and treatment of neuromyelitis optica spectrum disorders in clinical practice. Mult Scler Relat Disord. (2020) 45:102428. doi: 10.1016/j.msard.2020.102428 32763842

